# Self-Reported Digital Health Literacy and Work Engagement Among Nurses in UAE Hospitals

**DOI:** 10.3390/nursrep16050177

**Published:** 2026-05-20

**Authors:** Rasha Kadri Ibrahim, Noor Hafiz Saleem, Ruba Mohd Salameh, Amal Abdullah Alali, Bushra Ali Alnaqbi, Ahmed Yahya Ayoub

**Affiliations:** 1 Nursing Department, Fatima College of Health Sciences, Madinat Zayed P.O. Box 5788, United Arab Emirates; 2 Nursing Department, Fatima College of Health Sciences, Abu Dhabi P.O. Box 3798, United Arab Emirates; 3 Nursing Department, Sheikh Shakbout Medical City—Pure Health, Abu Dhabi P.O. Box 11001, United Arab Emirates

**Keywords:** health information technologies, computer literacy, information literacies, UAE hospitals, nurses, staff engagement, employee participation, work environment

## Abstract

**Aim:** This study aimed to evaluate self-reported digital health literacy levels and work engagement among nurses in the United Arab Emirates (UAE), while also examining associations with demographic factors and the interplay between digital health literacy and work engagement. **Background:** The integration of digital technologies into healthcare has transformed patient care, clinical practice, and administration. Nurses, as frontline practitioners, play a crucial role in utilizing digital tools to enhance patient interactions and navigate complex healthcare systems. **Methods:** Between May and August of 2024, 364 nurses in the United Arab Emirates participated in a cross-sectional design study. A standardized 21-item self-reported Digital Health Literacy questionnaire and a 9-item Utrecht Work Engagement Scale were administered. Descriptive statistics were used, with *t*-tests, ANOVA, correlations, and multiple linear regression applied. **Results:** The average score for self-reported digital health literacy (3.05 ± 0.57) and work engagement (4.83 ± 1.13) was high. Gender, age, work experience, and education level showed varying patterns of association with self-reported DHL and work engagement across total and subscale scores. Education level was significantly associated with self-reported DHL but not with work engagement. The overall work engagement score and its subscales were positively correlated with self-reported DHL. **Conclusions:** Our findings provide a robust basis for subsequent research on DHL and work engagement. These findings support the relevance of self-reported DHL as a factor associated with nurses’ work engagement in digitally intensive healthcare settings. The study reveals that nurses reported high levels of digital health literacy and work engagement.

## 1. Introduction

Digitization has dramatically improved patient care, clinical processes, and healthcare management in recent years [1]. Nurses are leading the digital revolution by utilizing technology to enhance patient outcomes and improve healthcare delivery. Nurses can directly engage with patients and manage complex digital health systems as frontline practitioners [1,2].

Work engagement is defined as a constructive, continuous, and accomplished mental state tied to a given job [3]. It is commonly assessed using a three-dimensional framework comprising vigor, dedication, and absorption. Vigor reflects high energy levels, mental resilience, and the willingness to devote effort to one’s work, even in the face of difficulties. Dedication indicates that an employee is intensely involved in their work, accompanied by a sense of meaningfulness, enthusiasm, and inspiration [4]. Absorption denotes being entirely concentrated and engrossed in work, often to the extent that time seems to pass quickly [3].

Research has shown that work engagement yields significant benefits for nurses, healthcare organizations, and patient care outcomes [5,6,7,8]. A study conducted in the United Arab Emirates found a significant positive correlation between job satisfaction and work engagement, with nurses reporting moderate satisfaction levels alongside high engagement scores [9]. A study conducted among 2369 nurses working in the UAE found that work engagement is associated with improved job performance, enhanced service delivery, and more effective task accomplishment, regardless of job demands. It also emphasizes the significance of job resources and organizational justice in improving employee engagement levels [10].

For healthcare organizations, enhanced engagement also contributes to broader organizational success, including increased efficiency, profitability, customer loyalty, and the ability to attract skilled professionals [11,12,13,14,15]. Recent papers documented a correlation between nurse involvement, decreased hospital patient mortality rates, and improved corporate profitability and performance [16,17]. Research has determined that the level of engagement exhibited by healthcare professionals, such as nurses, is directly correlated with the provision of excellent care and the safety of patients [18,19]. These findings underscore the need to enhance work engagement to promote nurse performance. They indicated positive ratings for the quality of care and patient safety measures [10,20].

As healthcare systems undergo rapid digital transformation, nurses are increasingly required to interact with complex digital tools and technologies. Digital Health Literacy (DHL) has emerged as a critical competency. In this study, DHL refers to nurses’ self-reported ability to access, understand, evaluate, and apply health information in the digital age [21,22,23]. Insufficient DHL may lead to stress, decrease proficiency, and reduce work engagement levels, highlighting the importance of digital competency in supporting workforce engagement [23,24,25,26,27,28].

In this study, DHL was selected as the key explanatory variable because it captures nurses’ perceived ability to access, understand, appraise, and apply digital health information in practice. Although DHL overlaps conceptually with related constructs such as digital competence, technology acceptance, and informatics readiness, these constructs emphasize different dimensions. Digital competence commonly refers to broader technical and professional capabilities, technology acceptance focuses on attitudes and behavioral intentions toward technology use, and informatics readiness reflects preparedness to adopt informatics systems. DHL was considered most appropriate for the present study because it focuses specifically on nurses’ self-reported ability to manage digital health information, which is directly relevant to everyday clinical work in digitally intensive hospital environments.

Enhancing DHL among nurses may be relevant to work engagement, patient outcomes, referral processes, and hospital operational efficiency [24,27,28]. Various studies have been conducted in this regard, both internationally and nationally. In a study by Cho et al. examining the relationship between electronic health literacy and health-promoting behaviors among South Korean hospital nurses, nurses with higher levels of electronic health literacy exhibited significantly more health-promoting behaviors [25]. Similarly, an institution-based cross-sectional study in Ethiopia revealed that Digital health is critical for seeking health information online to make informed decisions [24]. Furthermore, a cross-sectional study conducted in Jordan, involving 238 nurses, found that the level of DHL among nurses in Jordanian hospitals was highly satisfactory, particularly in terms of their proficiency in operational and navigational skills, as well as their ability to search for information [26]. A study in the UAE involving 551 nurses revealed median DHL scores of 70.2, indicating a moderate level of competence in accessing and interacting with digital health resources [22].

Conversely, nurses with insufficient DHL typically struggle to access medical information using technology and become wholly engrossed in subpar patient care; this disadvantage may also impair their communication skills when working in a multidisciplinary team, resulting in decreased work engagement [28]. Hillestad et al. have shown that negative perceptions of information systems negatively impact staff involvement in the digital environment, subsequently affecting patient care safety and quality [29]. A systematic review examined English-language studies on the DHLs of pharmacy employees and their computer skills training. The review found that hospital and community pharmacy professionals from Australia, Canada, and the United States do not have a clear understanding of DHL [30]. Similarly, a U.S.-based study of community hospital nurses emphasized the need for higher levels of computer literacy proficiency [31]. Gathering baseline data on nurses’ technological proficiency is necessary to assess their readiness to use digital health technologies [24]. This approach helps identify barriers and areas for improvement and supports the development of targeted strategies to enhance effective technology use [32].

**Figure 1:**
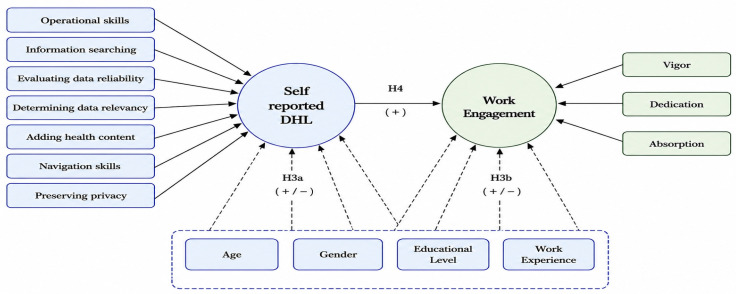
Conceptual model illustrating the hypothesized associations among self-reported digital health literacy, work engagement, and sociodemographic variables.

Drawing on the Job Demands–Resources (JD–R) model [33], this study conceptualizes self-reported DHL as a potential personal job resource in digitally intensive healthcare environments. According to the JD–R framework, job and personal resources may support employees’ capacity to manage work demands and are associated with motivational outcomes such as work engagement. In contemporary healthcare settings, nurses who perceive themselves as more capable of accessing, evaluating, and using digital health information may experience fewer difficulties when navigating digital workflows, which may be reflected in higher levels of vigor, dedication, and absorption. Therefore, this study hypothesized that self-reported DHL would be positively associated with overall work engagement and its dimensions. In addition to DHL, sociodemographic and professional characteristics, including age, gender, education level, and work experience, were incorporated into the conceptual model based on prior evidence indicating their associations with digital health literacy and work engagement among nurses. This theoretical framework supports the conceptual model illustrated in Figure 1.

Digital health technologies are extensively examined in healthcare literature; it is crucial to differentiate DHL, which denotes individuals’ perceived ability to access, navigate, evaluate, and utilize digital health information, from more general discussions regarding digital health infrastructure or technology adoption. Current research on nurses predominantly investigates digital instruments concerning efficiency, patient outcomes, or system usability, with limited studies addressing nurses’ DHL as an individual capability. Furthermore, whereas work engagement has been thoroughly investigated among nurses, research exploring the intersection of DHL with motivational and engagement-related outcomes is scarce. The majority of existing studies are from Western or East Asian contexts, with less evidence from the UAE and Middle East, where healthcare systems are undergoing rapid digitization and nurse workforces are notably multicultural.

The UAE presents a unique context, characterized by its rapid transformation in digital health, a diverse nursing workforce, and a strong national emphasis on healthcare innovation. Examining these contextual elements in a localized setting is crucial, as they may influence nurses’ perceptions and interactions with digital health tools. This gap highlights the necessity for context-specific research that rigorously investigates DHL and its correlation with work engagement among nurses in the UAE technologically advanced hospital environments. Figure 1 presents a conceptual model illustrating the hypothesized association between DHL, work engagement, and background variables informed by the Job Demands–Resources framework.

This research advances both the theoretical and practical dimensions of nursing and digital health literature. Theoretically, it extends the Job Demands–Resources model by examining self-reported DHL as a potential personal job resource associated with nurses’ engagement in technology-intensive work settings. Practically, the findings provide evidence-based insights for hospital administrators and nurse leaders in the UAE, underscoring the relevance of supporting nurses’ DHL as part of broader strategies to promote engagement, performance, and well-being.

This study identifies specific DHL categories that require support, including navigation skills and privacy preservation, to enable targeted training initiatives and organizational policies that optimize digital transformation in UAE hospitals.

The primary objectives of this study were

- To evaluate self-reported DHL and work engagement levels among nurses.
- To examine the relationship between demographic variables (age, sex, education level, and work experience) and nurses’ DHL and work engagement.
- To analyze the association between the self-reported DHL scale, the work engagement scale, and their respective subscales.

### Research Questions and Hypothesis

Research hypothesis.

**Hypothesis 1 (H1).**  *Nurses in SSMC and MZH settings will report a high level of self-reported DHL*.

**Hypothesis 2 (H2)**  *Nurses in SSMC and MZH settings will have a high level of work engagement*.

**Hypothesis 3 (H3)**  *There is a significant association between nurses’ sociodemographic variables and their self-reported DHL and work engagement*.

**Hypothesis 4 (H4)**  *There is a significant association between nurses’ self-reported DHL and work engagement*.

Research questions:

- What are the levels of self-reported DHL and work engagement among nurses working in the participating UAE hospitals?
- What are the associations between sociodemographic variables and hospital nurses’ DHL and work engagement?
- Is there an association between hospital nurses’ self-reported DHL and their work engagement?

## 2. Methods

### 2.1. Study Design

This study is cross-sectional, descriptive, multicenter, and correlational in design. Its reporting was based on the Strengthening the Reporting of Observational Studies in Epidemiology (STROBE) Statement checklist.

### 2.2. Setting and Participants

The research was conducted among nurses at Sheikh Shakhbout Medical City (SSMC) and Madinat Zayed Hospital (MZH) in the United Arab Emirates. SSMC is the largest tertiary hospital in the Emirate of Abu Dhabi. SSMC was established as part of Abu Dhabi’s Economic Vision 2030 to enhance healthcare services in the Emirate. Our world-class medical destination reinforces our vision for positioning Abu Dhabi as a global healthcare hub. It is a teaching hospital with a steady recruiting process. The hospital operates with a 522-bed capacity and is a leading healthcare institution in the United Arab Emirates. The hospital provides advanced care for patients with severe and complex conditions. SSMC has a vast nursing workforce and specialties such as critical care and emergency, oncology, medical and surgical, maternal and child health, stroke, surgical services, procedural areas, and outpatient clinics. MZH is part of AL Dhafra Hospitals, a member of the SEHA—Abu Dhabi Health Services Company network, which the Pure Health Group owns in the UAE. MZH provides a comprehensive range of services to both internal and external customers. MZH is a secondary healthcare entity that offers some select tertiary services. The licensed bed capacity is 158, with 105 currently in use. This number fluctuates according to the plan and the hospital’s surge capacity. Additionally, some of these beds are blocked due to maintenance or low patient volume; all services in MZH are based on accredited, evidence-based knowledge approved by the Department of Health.

The number of nurses participating in the study was determined using Epi-Info 7 software. The calculation considered a 5% margin of error, a 95% confidence level, 80% power, and a significance level of 0.05. The anticipated sample size was 380 nurses. The target population consisted of all registered nurses employed in the two participating institutions. A non-probability convenience sampling approach was used. All eligible registered nurses in the two participating hospitals who met the inclusion criteria were invited to participate through the online survey link, and those who agreed to participate completed the questionnaire. The inclusion criteria consisted of actively employed registered nurses at the two hospitals who had worked for a minimum of three months, completed their probation period, and were fully engaged in their roles. The study excluded nurse managers, supervisors, and registered nurses with less than three months of experience. All eligible registered nurses who met the inclusion criteria and were accessible during the data collection period were invited to participate through the online survey link. A total of 380 eligible nurses received the invitation, and 364 completed the questionnaire, yielding a response rate of 95.8% (364/380 × 100) between May and August 2024.

#### 2.2.1. Digital Health Literacy Scale (DHL)

This study employed a standardized, pre-designed questionnaire to examine nurses’ self-reported DHL. The questionnaire was designed to assess DHL level and has been previously validated in various demographics and regions [26]. The questionnaire has two components. The initial component comprises socio-demographic data, including factors such as gender, age, level of education, and work experience. The second half consists of 21 questions divided into seven subscales, each with three statements. The subscales encompass operational abilities (α = 0.894), information seeking (α = 0.912), evaluating reliability (α = 0.899), identifying relevance (α = 0.877), adding health content (α = 0.869), navigation abilities (α = 0.942), and safeguarding privacy (α = 0.956). The Likert scale used throughout this study has four response options, ranging from “very difficult” (ranked as 1) to “very easy” (ranked as 4). The Likert scale measures navigation abilities and safeguarding privacy subscales, with ratings ranging from “never” (1) to “always” (4). The total score for each subscale is calculated as the mean of the relevant items. The overall score on the scale ranges from 21 to 84, while each subscale yields a score between 3 and 12. Higher scores reflected higher self-reported digital health literacy, rather than objectively tested digital competence.

For each subscale, the percentage of the subscale total score was calculated by dividing the mean subscale score by the maximum possible score for that subscale and multiplying by 100. These percentages were interpreted using predefined thresholds: <20% very undesirable, 21–40% undesirable, 41–60% average, 61–80% desirable, and 81–100% very desirable. This classification method enables transparent interpretation and facilitates reproducibility across studies [26,34]. Prior validation studies reported acceptable reliability for the scale, ranging from 0.87 to 0.803 [34]. In the present study, the Cronbach’s alpha coefficient was 0.913.

#### 2.2.2. Work Engagement Tool

The Utrecht Work Engagement Scale (UWES) evaluates work engagement and is considered a short version [35]. This is scored on a seven-point Likert scale, ranging from 0 (representing ‘never’) to 6 (representing ‘always’), which measures three competencies: vigor (α = 0.836), dedication (α = 0.913), and absorption (α = 0.794), with three items allocated to each facet of interaction. The overall score and the subscale scores are calculated as means. The lowest score obtained from the scale is 0, while the highest score is 54; each subscale can yield a score between 0 and 18. Cutoff values were statistically defined to provide a precise interpretation of the derived scores for high (4–6), moderate (2 < 4), and low (0 < 2) reported engagements [36]. Prior validation studies reported excellent reliability for the scale, 0.965 [37]. In the present study, the Cronbach’s alpha coefficient was 0.901.

The cutoff points used to classify DHL and work engagement were based on a percentage-of-maximum scoring approach commonly applied in health and educational research using Likert-type scales. This approach facilitates standardized interpretation and comparability across subscales with identical scoring ranges and has been widely used in prior studies to describe relative levels of performance or perception [26,34,35].

#### 2.2.3. Validity & Reliability

The Digital Health Literacy Scale and the UWES-9 are established instruments with prior evidence of validity and reliability. In the present study, internal consistency reliability was assessed using Cronbach’s alpha. The Cronbach’s alpha coefficients were 0.913 for the Digital Health Literacy Scale and 0.901 for the Shortened Version of the UWES, indicating excellent internal consistency in the current sample. As this study was not designed as a full psychometric validation study, construct validity was interpreted based on prior validation evidence rather than a new validation model conducted in the present sample.

A pilot study was conducted with 36 nurses (10% of the overall sample size) to evaluate the clarity, applicability, relevance, and feasibility of the survey tools, as well as to estimate the time required for data collection. The pilot research results were not modified, and the nurses who participated in the pilot were included in the primary study population. This quantitative study employed procedural and statistical controls to prevent bias. Although convenience sampling was used, clear inclusion and exclusion criteria were applied to support consistency in participant selection. Verified and pre-tested measuring instruments ensured reliability and reduced measurement bias. To decrease social desirability biases, anonymity and confidentiality were highlighted.

To assess the potential influence of common method variance, Harman’s one-factor test was conducted separately for the DHL Scale and the UWES-9 because the two instruments measure different constructs. For each scale, all items were entered into an unrotated exploratory factor analysis using principal component analysis. The first factor accounted for 41.88% of the variance in the DHL Scale and 43.2% of the variance in the UWES-9, both below the commonly used 50% threshold. These results suggest that common method variance was unlikely to dominate the responses within each scale. However, Harman’s one-factor test is a limited diagnostic approach; therefore, the possibility of common method bias cannot be fully excluded.

### 2.3. Data Collection

The data collection for this study involved distributing survey questionnaires to nursing staff via email communication. Data were collected over four months, from 9 May 2024 to 31 August 2024, through the dissemination of an online questionnaire. This timeline was meticulously created to facilitate participant recruitment, questionnaire administration, and data collection, while maintaining data accuracy and quality. The questionnaire link, which took approximately 10–13 min to complete, was included in the email along with the principal investigator’s contact information. During the data collection period, the researchers were available to answer any questions participants may have had, ensuring that the study’s objectives were clear and understood.

### 2.4. Ethical Considerations

The Research Ethics Committee at Fatima College of Health Sciences, UAE, approved the ethical aspects of this study after reviewing the study protocol, instrument, and consent form presented to committee members. The authorization was granted on 17 April 2024 [IRB approval number: FECE-2-23-24 -RASHAIBRAHIM]. Furthermore, ethical approval was obtained from the SEHA Research Ethics Committee on 8 May 2024 (Approval number: SEHA-IRB-7380) and from Sheikh Shakhbout Medical City (Approval number: SSMCREC-490) on 4 July 2024. After being fully informed and receiving a detailed explanation of the study’s objective, the subject gave informed consent to participate. Confidentiality and anonymity were ensured by issuing a unique code number to each questionnaire. The nurses were told that their data would be kept confidential and used exclusively for research purposes. Participants were informed of their right to withdraw from the study at any time. We confirm that our study was conducted by the principles outlined in the Declaration of Helsinki.

### 2.5. Data Analysis

The current research data were processed using the Statistical Package for Social Sciences (SPSS) version 23. Descriptive data were quantified using numerical values, including numbers, percentages, minimum and maximum values, averages, and standard deviations. Data normality was assessed using the Shapiro–Wilk test which indicated no significant deviation from normality (*p* = 0.078). Accordingly, parametric statistical tests were applied for subsequent analyses.

Independent samples *t*-tests and one-way ANOVA were used to examine group differences in self-reported DHL and work engagement across categorical variables, while Pearson’s correlation coefficients were used to assess associations between continuous variables. Effect sizes were calculated to support interpretation of practical significance, with Cohen’s d used for independent samples *t*-tests, eta-squared (η^2^) for one-way ANOVA, and Pearson’s r interpreted as the effect size for correlation analyses. These univariate analyses were conducted to describe group differences and bivariate associations. Multiple linear regression analysis was then conducted to examine the independent association between self-reported DHL and work engagement after controlling for theoretically and empirically relevant sociodemographic variables. Predictor selection was guided by the Job Demands–Resources framework, prior empirical evidence, and conceptual relevance rather than solely by statistical significance in univariate analyses. Age, gender, education level, work experience, and self-reported DHL were entered into the adjusted model. Age and work experience were collected as continuous variables and categorized only for descriptive and group-comparison analyses. Their continuous values were entered into the multiple linear regression model. Gender was entered as a binary-coded variable. Education level was treated as an ordinal numeric variable according to its coded categories: diploma = 1, baccalaureate = 2, and higher education = 3.

Regression assumptions, including normality of residuals, homoscedasticity, and absence of multicollinearity, were assessed and met. Multicollinearity was assessed using tolerance and variance inflation factor (VIF) values. Results are presented as unstandardized coefficients (B) with standard errors, 95% confidence intervals, t-values, and *p*-values. Statistical significance was set at *p* ≤ 0.05 for all analyses.

## 3. Results

### 3.1. Participant Demographic Profile

The age range of 48.6% of the nurses who participated in the study is 31 to 40 years. The analysis revealed that 72.0% of the nurses were female, while 62.6% held a baccalaureate degree. According to the data, 27.2% of the participants had 11–15 years of work experience, while 25.3% had more than 20 years of experience (Table 1).

### 3.2. Self-Reported DHL and Work Engagement Among Nurses

On the DHL Scale, nurses received a mean score of 3.05 (SD = 0.57). This score indicates that nurses reported a high level of self-reported digital health literacy. Upon analyzing the mean scores for each subscale, it was found that the nurses achieved the highest scores in the “Operational skills” and “Information searching” subscales, with mean scores of 3.57 (SD = 0.54) and 3.37 (SD = 0.62), respectively. The subscales “Preserving privacy” and “Navigation skills” received the lowest mean scores, at 2.25 (SD = 1.16) and 2.40 (SD = 1.10), respectively (Table 2).

The average score for the Work Engagement Scale was 4.83 (SD = 1.13), given a possible range of 0–6. This mean score indicates that, on average, the nurses perceived their engagement level as high. Upon examining the subscales, we observe similar trends. The mean score for the Absorption subscale was 4.94 (SD = 1.12), dedication 4.86 (SD = 1.25), and the ’Vigor’ subscale was 4.69 (SD = 1.27) (Table 2).

### 3.3. Associations Between Sociodemographic Variables, DHL, and Work Engagement

According to Table 3, there were statistically significant differences between male and female nurses regarding the DHL total score, preserving privacy, and navigation skills subscales (*p* < 0.05). Significant gender differences were also observed in the total UWES score and in the vigor and absorption subscales (*p* < 0.05). Male nurses achieved superior results in these specific areas (Table 3).

A statistically significant difference was observed between the age variable and work experience variable in the total score of the digital health scale, as well as the subscales “Preserving privacy” and “Navigation skills.” Similarly, the total score and all subscales of the Work Engagement Scale showed significant differences (*p* < 0.05). Nurses aged 40 and over got higher scores, and those with over 20 years of experience performed better in these areas than their counterparts (Table 3). Regarding educational level, no significant differences were detected between the work engagement scale and its subscales. However, a statistically significant difference was noticed between the educational level variable and the DHL Scale Total, “Information searching,” “Evaluation data reliability,” “Determining data relevancy,” and “Adding health content” subscales (*p* < 0.05). Nurses with more qualifications received higher scores in those categories.

### 3.4. Associations Between DHL and Work Engagement

A small to moderate, positive, and statistically significant association was observed between the overall work engagement scale and its domains, as well as the overall DHL and its domains (Table 4).

### 3.5. Association Between DHL and Work Engagement After Controlling for Sociodemographic Variables

A multiple linear regression analysis (Table 5) was conducted to examine the independent association between self-reported DHL and work engagement after controlling for age, gender, education level, and work experience. The overall model was significant (F = 24.433, *p* < 0.001) and explained approximately 25.4% of the variance in work engagement (R^2^ = 0.254; adjusted R^2^ = 0.244). Tolerance values exceeded 0.10 and variance inflation factor (VIF) values were below 5 for all predictors, indicating that multicollinearity was not a concern in the regression model. After adjustment, self-reported DHL remained significantly and positively associated with work engagement (B = 0.340, 95% CI [0.261, 0.419], *p* < 0.001). Work experience was also positively associated with work engagement (B = 0.627, 95% CI [0.367, 0.887], *p* < 0.001), whereas age showed a negative association (B = −0.552, 95% CI [−0.820, −0.284], *p* < 0.001). Education level was not significantly associated with work engagement in the adjusted model (B = −1.028, 95% CI [−2.606, 0.551], *p* = 0.201), and gender was not significant (B = −1.634, 95% CI [−3.679, 0.412], *p* = 0.117). Overall, these findings suggest that self-reported DHL was associated with work engagement beyond selected sociodemographic characteristics.

## 4. Discussion

This research investigated self-reported DHL and work engagement among nurses in hospitals across the UAE, and analyzed their relationships with sociodemographic factors, based on the Job Demands–Resources framework. The discussion is structured around the study hypotheses, initially focusing on overall levels of DHL and work engagement, followed by engagement dimensions, and ultimately evaluating the independent contribution of DHL while controlling for demographic variables.

### 4.1. DHL and Overall Work Engagement

Consistent with Hypothesis 1, the results demonstrate that nurses employed in hospitals in the UAE exhibited predominantly high levels of DHL, especially in the areas of operational skills and information searching. These findings indicate that nurses perceived themselves as competent in executing routine digital tasks and retrieving health-related information, which may reflect the technologically advanced infrastructure of healthcare facilities in the UAE and nurses’ regular interaction with electronic health systems. This may be ascribed to an intersection of factors, including lifelong learning [36], access to digital health technologies [37], and organizational support [38]. Comparable patterns have been documented in research conducted in Iran and Kuala Lumpur, where healthcare personnel exhibited acceptable to high levels of digital health literacy within operational domains [34,39].

However, despite these strengths, navigation skills and privacy protection showed as comparatively weaker subdomains, constituting a significant and somewhat unanticipated gap considering the overall high DHL scores. The likely explanation for this observation is the rapid development of digital technologies in UAE hospitals, which may necessitate frequent system updates and modifications without equivalent structured training [40]. Furthermore, the complexity of privacy settings, which is perceived as an organizational responsibility rather than an individual competency, potentially reduces nurses’ confidence in this area. This finding partially diverges from studies in Jordan and some Western contexts, where navigation and privacy skills were reported as stronger components of digital literacy among nurses [26,41,42,43].

From a Job Demands–Resources (JD–R) perspective, these findings indicate that self-reported digital health literacy may serve as a personal job resource. However, deficiencies in navigation skills and privacy protection may hinder nurses’ capacity to fully utilize digital systems and could elevate cognitive load in technology-intensive settings. Addressing these specific DHL deficiencies is therefore essential, as they compromise the potential motivational advantages of digital tools, even in environments characterized by high levels of digital maturity. Collectively, these findings underscore that digital health literacy among nurses varies across different domains and underscore the need for domain-specific DHL training rather than generalized digital education, ensuring that nurses are adequately equipped to function confidently and safely within increasingly complex digital healthcare environments.

### 4.2. DHL and Engagement DimensionsTop of FormBottom of Form

Consistent with Hypothesis 2, nurses in the present study reported generally high levels of work engagement, with absorption emerging as the most prominent dimension, followed by dedication and vigor. This trend suggests that nurses were highly immersed in their work, even when energy levels (vigor) were comparatively lower. Similar engagement profiles have been reported among nurses in the UAE and other healthcare systems, where absorption often dominates engagement dimensions due to the demanding and task-focused nature of nursing work [10]. This is explained by the idea that nurses operating within contemporary healthcare environments tend to demonstrate a robust professional identity and a heightened sense of consistency, which are associated with increased work engagement. Furthermore, working in a dynamic and demanding ward necessitates complete engagement in the assigned tasks. This interpretation aligns with previous research indicating that elevated absorption levels [12,35,44].

Notably, although all engagement dimensions were positively associated with DHL, the strength of these associations was small to moderate, indicating that digital literacy is regarded as one of multiple contributing factors rather than the primary driver of engagement. A multi-level review revealed that the digital era introduces new variables that affect engagement, such as platform tools, algorithmic control, and virtual collaboration. These factors can reshape how engagement is experienced and measured, potentially weakening the direct association with DHL [45,46].

However, the relatively weaker association with vigor suggests that improving digital skills alone may be insufficient to enhance nurses’ energy and resilience without parallel organizational support. These findings support the Job Demands–Resources framework, emphasizing that digital competencies should be complemented by supportive work environments, adequate staffing, and well-designed digital systems to foster balanced and sustainable engagement.

### 4.3. DHL in Relation to Work Engagement After Controlling for Demographics

The observed demographic variations are considered reasonable patterns supported by prior research and theoretical frameworks, rather than conclusive explanations. In partial support of Hypothesis 3, several sociodemographic characteristics, namely age, gender, and work experience, were significantly associated with both DHL and work engagement. Nurses in mid-career stages and those with longer professional experience demonstrated higher levels of digital health literacy and engagement, reflecting the accumulation of resources over time, such as emotional regulation and career identity, which enhance engagement. Employees with more work experience tend to demonstrate greater absorption and dedication in their roles [47]. These findings are consistent with studies conducted in Iran and Ethiopia, which similarly reported higher DHL and engagement-related outcomes among more experienced nurses [24,34].

After controlling for sociodemographic variables, digital health literacy remained positively and independently associated with work engagement, supporting Hypothesis 4 and reinforcing the study’s conceptual model, which is grounded in the Job Demands–Resources framework. This suggests that DHL functions as a personal job resource that covaries with engagement in digitally intensive nursing environments. Nurses with higher DHL may be better equipped to navigate digital workflows efficiently, potentially reducing cognitive strain and supporting sustained engagement.

Notably, educational level was significantly associated with self-reported DHL but not with work engagement, representing an important non-significant but informative finding. This suggests that formal education may support nurses’ perceived ability to access, evaluate, and use digital health information but may not directly translate into higher engagement. Work engagement may be more strongly influenced by experiential, organizational, and contextual factors, such as role demands, workload, staffing adequacy, leadership support, organizational support, and the quality of the work environment. In addition, highly educated nurses may have greater expectations regarding role autonomy, career progression, professional recognition, and access to supportive work-environment resources. If these expectations are not fully met, the potential engagement benefits associated with advanced education may be offset. Similar patterns have been reported in prior studies. For instance, a study on Mexican workers found no significant differences in work engagement based on educational attainment [48]. The Job Demands–Resources model also emphasizes that job resources and demands, rather than educational level alone, are more proximal predictors of work engagement [49].

The different direction of the age finding between the univariate and adjusted analyses should be interpreted in relation to work experience. Although nurses aged 40 years and above reported higher engagement in the univariate analysis, age showed a negative association in the adjusted model after controlling for work experience, education level, gender, and self-reported DHL. This may reflect shared variance between age and work experience, where work experience captures accumulated professional resources, such as clinical confidence, role familiarity, emotional regulation, and professional identity, more directly than chronological age. Therefore, the negative coefficient for age should be interpreted cautiously and not as evidence that older age alone reduces engagement.

Gender differences were also observed, with male nurses showing higher DHL and engagement scores in some domains. However, these findings should be interpreted cautiously, as gender-related differences in engagement are inconsistently reported across the literature and may reflect cultural or organizational factors rather than stable individual characteristics. Overall, the persistence of the DHL and engagement association after adjustment for demographic factors underscores the relevance of digital health literacy within contemporary nursing practice, while the cross-sectional design necessitates an associative rather than causal interpretation.

## 5. Conclusions

This study identified high levels of self-reported DHL and work engagement among nurses employed in hospitals in the UAE. Small to moderate positive associations were found between DHL and total work engagement, as well as its dimensions, suggesting that perceived digital health literacy and engagement were related in this sample. Operational skills and information search were the most significant components of DHL, whereas absorption showed the highest engagement levels. Sociodemographic characteristics, such as gender, age, and work experience, were associated with differences in DHL and work engagement, but education had a negligible association with engagement. These findings summarize the association between self-reported digital health literacy and work engagement in a digitally intensive nursing environment.

## 6. Implications

The results have significant implications for healthcare administration and nursing practice in the UAE. The positive association between self-reported DHL and work engagement indicates that healthcare organizations could gain from investing in organized and ongoing digital health training programs customized to the varied skill levels and linguistic backgrounds of the multicultural nursing workforce. The relatively lower results in navigation skills suggest a necessity for experiential, practice-oriented training, including guided workshops and simulation sessions centered on using electronic health records, clinical dashboards, and digital decision-support tools. Engaging nurses in the development of digital solutions and fostering peer learning may enhance participation and diminish opposition to technology adoption.

The lowered performance in privacy protection highlights the necessity of enhancing nurses’ proficiency in digital ethics and data security. Specialized training on patient confidentiality, cybersecurity awareness, and the secure management of digital information could be incorporated into ongoing professional development programs. Healthcare organizations can improve engagement by cultivating a supportive digital culture via accessible IT assistance, digital well-being policies, and sustainable workloads. Considering the variety of the UAE healthcare workforce, implementing culturally and linguistically inclusive digital strategies and setting benchmarks to assess the impact of digital health technology on nurse engagement may facilitate sustainable and equitable digital integration.

### Strengths and Limitations

This research possesses numerous strengths. Initially, it encompassed a substantial sample of nurses, hence augmenting the reliability of the results. Secondly, validated and widely used measures were employed to evaluate DHLs and work engagement, thus reinforcing the reliability of the findings. The article examines an under-explored context, offering empirical evidence from nurses in UAE healthcare environments, where data is scarce.

Several limitations must be acknowledged. The cross-sectional approach restricts the capacity to establish causal inferences between DHL and work engagement. In addition, the regression model explained approximately 25% of the variance in work engagement, indicating that other individual, organizational, and contextual factors may also contribute to nurses’ engagement. Future studies employing longitudinal or intervention-based methodologies would yield a deeper understanding of the changing patterns of work engagement and the impact of sustained DHL development on this engagement. Furthermore, the dependence on self-reported data presents the possibility of response bias, as participants may exaggerate or minimize their perceptions. Future research could enhance its rigor by including objective indicators, such as digital system usage logs or performance measurements, alongside self-reported assessments. Moreover, the use of convenience sampling from two participating hospitals may limit the generalizability of the findings to nurses in other UAE healthcare settings. To improve external validity, future research should incorporate multi-site studies with a more representative sample from various healthcare institutions and geographies.

## Figures and Tables

**Table 1:** Participant Demographic Profile (n = 364).

Variables	n	%
**Gender**		
Male	102	28.0
Female	262	72.0
**Age years**		
20–30 years	55	15.1
31–40 years	177	48.6
40–50 years	132	36.3
**Education level**		
Diploma	41	11.3
Baccalaureate	228	62.6
Higher education	95	26.1
**Work experience**		
0–5 years	46	12.6
6–10 years	47	12.9
11–15 years	99	27.2
16–20 years	80	22.0
>20 years	92	25.3

**Table 2:** Total and subscale mean scores of digital health literacy and shortened version of Utrecht work engagement for nurses (n = 364).

Scales and Their Subscales	Mean ± SD	% of Subscale Total Score	Level
**Digital Health Literacy Scale**	3.05 ± 0.57	68.30	Desirable
Operational skills	3.57 ± 0.54	85.60	Very desirable
Information searching	3.37 ± 0.62	79.00	Desirable
Evaluation data reliability	3.21 ± 0.70	73.60	Desirable
Determining data relevancy	3.27 ± 0.62	75.60	Desirable
Adding health content	3.27 ± 0.60	75.60	Desirable
Navigation skills	2.40 ± 1.10	46.60	Average
Preserving privacy	2.25 ± 1.16	41.60	Average
**Shortened Version of UWES**	4.83 ± 1.13	80.50	High
Vigor	4.69 ± 1.27	78.10	High
Dedication	4.86 ± 1.25	81.00	High
Absorption	4.94 ± 1.12	82.30	High

**Table 3:** Comparison of the Characteristics of Nurses with the DHL Scale and Shortened Version of UWES for Nurses (n = 364).

Characteristics		Digital Health Literacy Scale (DHL)								UWES-9			
		Operational Skills	Information Searching	Evaluation Data Reliability	Determining Data Relevancy	Adding Health Content	Navigation Skills	Preserving Privacy	Overall	Vigor	Dedication	Absorption	Overall
**Gender**	Male	10.69 ± 1.45	10.20 ± 1.71	9.72 ± 2.12	9.87 ± 1.89	9.84 ± 1.90	8.17 ± 3.35	7.82 ± 3.33	66.30 ± 11.81	14.89 ± 3.45	15.16 ± 3.35	15.63 ± 2.77	45.68 ± 8.73
	Female	10.72 ± 1.68	10.06 ± 1.90	9.60 ± 2.10	9.81 ± 1.86	9.81 ± 1.78	6.82 ± 3.21	6.32 ± 3.47	63.13 ± 11.92	13.76 ± 3.89	14.35 ± 3.87	14.50 ± 3.51	42.62 ± 10.54
	t	0.17	0.64	0.46	0.30	0.16	3.56	3.77	2.29	2.57	1.84	3.21	2.60
	*p*	0.869	0.520	0.647	0.758	0.873	<0.001	<0.001	0.023	0.010	0.067	0.001	0.010
**Age years**	20–30 years	10.76 ± 1.76	10.09 ± 1.68	9.67 ± 2.21	9.80 ± 1.88	10.04 ± 1.67	6.67 ± 3.32	6.53 ± 3.34	63.56 ± 10.80	14.42 ± 3.62	14.96 ± 3.37	15.38 ± 2.94	44.76 ± 9.02
	31–40 years	10.62 ± 1.51	9.92 ± 1.86	9.46 ± 2.04	9.73 ± 1.72	9.73 ± 1.65	6.79 ± 3.25	6.34 ± 3.32	62.60 ± 11.10	13.54 ± 3.94	13.99 ± 4.08	14.27 ± 3.54	41.80 ± 10.62
	40–50 years	10.80 ± 1.70	10.33 ± 1.88	9.85 ± 2.13	9.95 ± 2.04	9.85 ± 2.07	7.95 ± 3.25	7.36 ± 3.71	66.11 ± 13.25	14.65 ± 3.60	15.21 ± 3.31	15.32 ± 3.17	45.18 ± 9.62
	F	0.51	1.89	1.28	0.53	0.63	5.64	3.42	3.34	3.53	4.45	4.69	4.81
	*p*	0.600	0.152	0.279	0.588	0.532	0.004	0.034	0.037	0.030	0.012	0.010	0.009
**Education level**	Diploma	10.56 ± 1.45	9.44 ± 1.87	8.76 ± 2.20	8.71 ± 1.81	8.93 ± 1.88	6.27 ± 2.80	5.73 ± 2.83	58.39 ± 9.96	14.32 ± 3.58	15.00 ± 3.44	14.93 ± 3.20	44.24 ± 9.14
	Baccalaureate	10.59 ± 1.75	10.08 ± 1.91	9.70 ± 2.16	9.94 ± 1.88	9.88 ± 1.85	7.37 ± 3.41	6.96 ± 3.62	64.52 ± 12.36	13.97 ± 4.10	14.33 ± 4.07	14.69 ± 3.57	42.99 ± 11.06
	Higher education	11.05 ± 1.30	10.42 ± 1.61	9.86 ± 1.83	10.03 ± 1.69	10.05 ± 1.57	7.17 ± 3.20	6.65 ± 3.37	65.24 ± 11.20	14.22 ± 3.09	15.00 ± 2.95	15.08 ± 2.86	44.31 ± 8.08
	F	2.94	4.15	4.32	8.74	6.07	1.96	2.19	5.36	0.23	1.37	0.49	0.69
	*p*	0.054	0.017	0.014	<0.001	0.003	0.143	0.113	0.005	0.792	0.256	0.614	0.500
**Work experience**	0–5 years	10.89 ± 1.66	9.89 ± 1.64	9.50 ± 1.99	9.52 ± 1.81	9.57 ± 1.92	5.78 ± 2.66	5.30 ± 2.54	60.46 ± 8.61	12.74 ± 3.98	13.35 ± 3.96	14.13 ± 3.28	40.22 ± 10.14
	6–10 years	10.17 ± 1.67	9.81 ± 1.39	9.60 ± 1.36	9.89 ± 1.29	9.74 ± 1.42	6.49 ± 3.11	6.11 ± 3.42	61.81 ± 10.34	12.98 ± 4.26	13.70 ± 4.39	13.21 ± 4.35	39.89 ± 12.15
	11–15 years	10.67 ± 1.52	9.87 ± 2.06	9.21 ± 2.26	9.64 ± 1.80	9.70 ± 1.64	6.72 ± 3.24	6.22 ± 3.28	62.02 ± 11.24	13.93 ± 3.44	14.42 ± 3.53	14.72 ± 2.96	43.07 ± 8.93
	16–20 years	10.91 ± 1.37	10.48 ± 1.69	9.99 ± 2.07	10.01 ± 1.83	10.09 ± 1.63	7.68 ± 3.47	7.36 ± 3.60	66.51 ± 12.09	14.84 ± 3.70	15.18 ± 3.57	15.59 ± 2.89	45.60 ± 9.49
	>20 years	10.76 ± 1.83	10.26 ± 1.99	9.87 ± 2.27	9.98 ± 2.20	9.88 ± 2.22	8.36 ± 3.20	7.79 ± 3.69	66.90 ± 13.78	14.80 ± 3.65	15.29 ± 3.46	15.42 ± 3.31	45.52 ± 10.04
	F	1.82	1.84	1.92	0.93	0.82	6.85	5.92	4.485	4.24	3.35	5.23	4.69
	*p*	0.124	0.120	0.106	0.446	0.511	<0.001	<0.001	0.002	0.002	0.010	<0.001	0.001

SD: Standard deviation, t: Student *t*-test, F: One-way analysis of variance (ANOVA), *p* ≤ 0.05 indicates statistical significance.

**Table 4:** The relationship between the DHL Scale and the UWES-9 for Nurses (n = 364).

Scales and Subscales		The UWES-9			
DHL Scale		Vigor	Dedication	Absorption	Overall
Operational skills	r	0.36	0.26	0.31	0.34
	*p*	<0.001	<0.001	<0.001	<0.001
Information searching	r	0.24	0.23	0.24	0.25
	*p*	<0.001	<0.001	<0.001	<0.001
Evaluation of data reliability	r	0.25	0.22	0.23	0.25
	*p*	<0.001	<0.001	<0.001	<0.001
Determining data relevancy	r	0.24	0.21	0.21	0.24
	*p*	<0.001	<0.001	<0.001	<0.001
Adding health content	r	0.37	0.34	0.36	0.39
	*p*	<0.001	<0.001	<0.001	<0.001
Navigation skills	r	0.38	0.32	0.36	0.38
	*p*	<0.001	<0.001	<0.001	<0.001
Preserving privacy	r	0.42	0.32	0.38	0.40
	*p*	<0.001	<0.001	<0.001	<0.001
Overall	r	0.45	0.38	0.42	0.45
	*p*	<0.001	<0.001	<0.001	<0.001

r: Pearson coefficient, *p* ≤ 0.05 indicates statistical significance.

**Table 5:** Multiple linear regression predicting work engagement (n = 364).

	B	SE	*t*	*p*	CI (LL–UL)
constant	37.470	5.055	7.413	<0.001 *	27.530–47.410
Age	−0.552	0.136	−4.052	<0.001 *	−0.820–−0.284
Work Experience	0.627	0.132	4.735	<0.001 *	0.367–0.887
Gender (Female)	−1.634	1.040	−1.571	0.117	−3.679–0.412
Educational Level	−1.028	0.803	−1.280	0.201	−2.606–0.551
Self-reported DHL	0.340	0.040	8.475	<0.001 *	0.261–0.419
R^2^ = 0.254, adjusted R^2^ = 0.244, SE = 8.822, F = 24.433 *, *p* < 0.001 *					

B = unstandardized coefficient; SE = standard error; CI = confidence interval, LL: Lower limit, UL: Upper Limit. Age and work experience were entered into the model as continuous variables. * *p* ≤ 0.05 indicates statistical significance.

## Data Availability

The data that support the findings of this study are not publicly available due to confidentiality and ethical restrictions but are available from the corresponding author upon reasonable request and with appropriate approvals.

## Public Involvement Statement

Patients or the public were not involved in the design, conduct, reporting, or dissemination of this research.

## Guidelines and Standards Statement

This manuscript was drafted and reported in accordance with the Strengthening the Reporting of Observational Studies in Epidemiology (STROBE) Statement guidelines for cross-sectional observational research [50].

## Abbreviations

DHL	Digital Health Literacy
UWES	Utrecht Work Engagement Scale
SSMC	Sheikh Shakbout Medical City
MZH	Madinat Zayed Hospital
UAE	United Arab Emirates
